# Transcriptional biomarker discovery toward building a load stress reporting system for engineered *Escherichia coli* strains

**DOI:** 10.1002/bit.28567

**Published:** 2023-10-09

**Authors:** Yiming Huang, Anil Wipat, Jaume Bacardit

**Affiliations:** ^1^ Interdisciplinary Computing and Complex BioSystems Group Newcastle University Newcastle upon Tyne UK

**Keywords:** biomarker discovery, machine learning, metabolic load, system and synthetic biology, transcriptomics

## Abstract

Foreign proteins are produced by introducing synthetic constructs into host bacteria for biotechnology applications. This process can cause resource competition between synthetic circuits and host cells, placing a metabolic burden on the host cells which may result in load stress and detrimental physiological changes. Consequently, the host bacteria can experience slow growth, and the synthetic system may suffer from suboptimal function. To help in the detection of bacterial load stress, we developed machine‐learning strategies to select a minimal number of genes that could serve as biomarkers for the design of load stress reporters. We identified pairs of biomarkers that showed discriminative capacity to detect the load stress states induced in 41 engineered *Escherichia coli* strains.

## Graphical abstract

This study identifies transcriptional biomarkers that can detect load stress in *Escherichia coli*. By analyzing RNA‐seq data collected in heterologous gene expression cases and various conditions, the authors developed an ensemble of machine learning models to identify a minimal number of biomarkers discriminating load stress states from other cellular states. Three pairs of biomarker genes are thoroughly characterised for their biological significance.

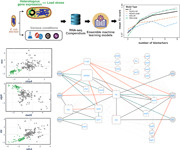

## 1 INTRODUCTION

In the biotechnology industry, prokaryotic expression systems are utilized to yield valuable products such as enzymes, chemicals, pharmaceuticals, and biofuels. *Escherichia coli* remains the preferred host strain to be engineered for the generation of diverse proteins (Castineiras et al., 2018; Gupta & Shukla, 2016). Most of these proteins are not naturally found in *E. coli*, thus synthetic constructs are designed and inserted to enable the introduction of foreign genes and proteins in the host strain (Burgess‐Brown et al., 2021). Unfortunately, the expression of the synthetic constructs can excessively consume the cellular resources (Gyorgy et al., 2015; Shachrai et al., 2010) and impose an unnatural metabolic burden on the bacteria (Wu et al., 2016). The load stress resulting from this metabolic burden can trigger detrimental physiological adaptations that potentially harm not only the growth of the host bacteria (Borkowski et al., 2016) but also the performance of the synthetic system as a whole (Cardinale & Arkin, 2012; Kim et al., 2020; Tan et al., 2009). Therefore, understanding the host strain's reaction to metabolic load and monitoring the cellular load stress are essential for successful synthetic construct expression.

Bacteria grow in environments that are constantly changing, often subjecting them to periods of multifarious stress conditions. As a result, they have evolved mechanisms to induce gene expression, metabolic and physiological changes that can help mitigate the damage resulting from these stresses (Gottesman, 2019; Jozefczuk et al., 2010). Transcriptomics technologies, such as RNA‐seq (Creecy & Conway, 2015), have revolutionized our ability to study bacterial transcriptomes in different conditions, allowing us to investigate how they shift in response to changing environments and infer the gene regulatory parts involved in stress responses (Goswami & Rao, 2018; Roncarati & Scarlato, 2017). Focusing on the load stress that the engineered strains can undergo while heterologous proteins expression, some studies have explored the transcriptional changes in host cells and identified key biomarker genes that react to load stress (Dürrschmid et al., 2008; Sharma et al., 2011). Additionally, feedback control systems incorporating these reporting genes or promoters have been developed to recognize load stress states and adjust synthetic construct expression (Ceroni et al., 2015, 2018; McBride et al., 2021).

These previous load stress studies, however, have looked at only a few synthetic constructs and a small number of foreign proteins, leading to restricted understanding of the host response to load stress. The resulted load stress reporting systems in these studies may therefore fail to recognize the cellular states induced by various unseen foreign protein expression. Meanwhile, these prior research have been also limited on transcriptional changes to load stress, missing comparison between load stress and other stress states (e.g., heat, acid, nutrients scarcity) in the host cells which are also common in industrial production (Tao et al., 2022; Yang et al., 2021). The biomarker genes identified in these cases are thus not unique to load stress and can mistakenly detect other stress responses that cause similar expression shifts in these load stress‐reporting genes.

This study aimed to develop machine learning methods for pinpointing a few key genes in *E. coli* that can discriminate load stress state induced by expressing a larger set of heterologous genes, with respect to a wide range of other cellular states presented by growing in various environments. We hypothesized that a minimal number of genes can be identified to indicate load stress by mining a large‐scale transcriptome that contains both samples induced with many different heterologous genes and samples grown in assorted conditions. A recent compendium of *E. coli* RNA‐seq data Precise2 (Lamoureux et al., 2021) is such a transcriptomics data set, on which we studied the transcriptional response to load stress and developed an ensemble of feature selection models to optimally decide the number of genes required to sense load stress. We highlighted the biomarker genes with great prediction performance and discriminative power. The identified biomarker genes can be harnessed to improve the performance of the burden feedback systems to monitor and relieve the load stress states elicited by producing a wide range of foreign proteins in *E. coli* cells.

## 2 MATERIALS AND METHODS

### 2.1 RNA‐seq data acquisition and normalization

PRECISE 2.0 (Lamoureux et al., 2021) is a compendium of RNA‐seq profiles, available at https://github.com/SBRG/precise2, for *E. coli* K‐12. This data set is suitable for exploring the load stress transcriptional response because it collects transcriptomes from a large‐scale heterologous gene expression experiment and many other experiments applying gene and environmental perturbations while growing the bacteria. To identify transcriptional biomarkers for load stress state in *E. coli*, we analyzed 251 gene expression profiles from this data set. These include (a) 169 samples of MG1655 strain grown with various environmental changes such as alternative carbon sources, alternative nitrogen sources, nutrient limitation, antibiotic drugs, anaerobic condition, and other stimulus (e.g., acid, oxidative drugs, ethanol, and NaCl) and (b) 82 samples of 41 engineered strains which respectively expressed a heterologous gene or had an empty plasmid inserted.

Lamoureux et al. processed the raw data using a Nextflow pipeline (https://github.com/avsastry/modulome-workflow) designed for microbial RNA‐seq datasets and reported read counts and log‐transformed Transcripts per Million (log‐TPM) after quality control. We used the gene expression read counts for the differential expression analysis. For biomarker discovery study with feature selection methods, we further normalized the log‐TPM to minimize the between‐experiments noises by subtracting study‐specific reference expression quantities. The study‐specific reference expression quantities were calculated as the average expression levels of control samples from the same study wherever possible. In the studies where the control experiments were not conducted, the reference expression quantities were computed from the samples grown with least environmental changes due to different base medium or carbon sources. Please find the reference sample list and normalized gene expression quantities in Supporting Information: Table S1.

### 2.2 Identifying differentially expressed genes (DEGs) and overrepresented gene ontology (GO) terms

The R package DESeq2 (Love et al., 2014) was applied to identify DEGs on 251 gene expression profiles collected in various conditions. We compared pairs of sample groups defined by 53 environmental changes (Supporting Information: Table S2a), including heterologous gene expression conditions that can induce load stress states, treatments of different stimuli, alternative nutrients or medium bases, nutrients limitation, anaerobic growth, and antibiotic addition. The significance threshold of DEGs was set as log‐fold‐change>1 and p<0.05 (obtained by Wald statistical test with multiple testing corrected by the Benjamini and Hochberg method).

GO enrichment analyzes were performed to study the overrepresented functions in these DEGs. Fisher exact test with Bonferroni correction was used to select the GO items with false discovery rate less than 0.01.

### 2.3 Regulon and iModulon activity analysis

A regulon is a group of genes or operons that are turned on or off in response to the same signal by the same regulatory protein. The regulons used in this study were taken from an *E. coli* transcriptional regulatory network which integrates RegulonDB v10.5 (Santos‐Zavaleta et al., 2019), Ecocyc (Keseler et al., 2021) and a recent study about uncharacterized transcriptional factors (Gao et al., 2021). This regulatory network consists of 371 regulons and 3407 targeted genes.

An iModulon, which was coined by Sastry et al. (2019), is a group of genes representing an independently modulated signal that are likely controlled by the same or related regulators. This concept can be mathematically represented as **X** = M*A, where $X_{\left(n,m\right)}$ represents the expression level of ${\mathrm{gene}}_{n}$ in ${\mathrm{sample}}_{m}$. **M** connects genes to iModulons—$M_{\left(n,j\right)}$ assigns a weight to ${\mathrm{gene}}_{n}$ for ${\mathrm{iModulon}}_{j}$. **A**connects iModulons to biological samples– $A_{\left(j,m\right)}$ indicates the activity level of ${\mathrm{iModulon}}_{j}$ in ${\mathrm{sample}}_{m}$. Therefore, iModulons can be inferred from large‐scale transcriptomic data to suggest hypothetical regulatory mechanisms in addition to prior known regulons. Here we analyzed the iModulons generated by Lamoureux et al. (2021) from PRECISE2 transcriptomics data for *E. coli*. The total of 218 iModulons are listed and characterized in an *E. coli* iModulon database https://imodulondb.org/search.html?organism=e_coli&dataset=precise2.

In addition to performing a differential expression analysis across 53 environmental changes at the gene level, we conducted a comprehensive quantification of regulon and iModulon activities. This involved assessing the activity level of each regulon or iModulon by calculating both a percentage index and an intensity index. As defined in Equation (1), the percentage index of a given regulon (*i*) or iModulon (*j*) in response to an environmental (*k*) demonstrates the proportion of DEGs that are matched within this regulon or iModulon. The intensity index, however, was calculated differently for regulons and iModulons (Equation 2). For a specific regulon (*i*) in an environmental change (*k*), its intensity index was determined by calculating the average log‐fold‐change (LFC) of the corresponding DEGs that overlapped with this regulon. For a specific iModulon (*j*) in an environmental perturbation (*k*), its intensity index simply indicates the log‐fold‐change of this iModulon—the expression level of an iModulon in a specific condition represents the weighted summation of the entire gene set's expression.

(1) $$\begin{array}{ccc} {\mathrm{Percentage}}_{\left(i,k\right)} & = & \frac{|\cap \left({\mathrm{regulon}}_{i},{\mathrm{DEGs}}_{k}\right)|}{|\mathrm{DEG}s_{k}|},\\ {\mathrm{Percentage}}_{\left(j,k\right)} & = & \frac{|\cap \left({\mathrm{iModulon}}_{j},{\mathrm{DEGs}}_{k}\right)|}{|{\mathrm{DEGs}}_{k}|}, \end{array}$$

(2) $$\begin{array}{ccl} intensity_{\left(i,k\right)} & = & \mathrm{mean}\left({\mathrm{LFC}}_{\left({\mathrm{gene}}_{n},k\right)}|{\mathrm{gene}}_{n}\in \cap \left({\mathrm{regulon}}_{i},{\mathrm{DEGs}}_{k}\right)\right)\\ {\mathrm{intensity}}_{\left(j,k\right)} & = & LFC_{\left(iModulon_{j},k\right)} \end{array}.$$

### 2.4 Feature selection methods

To identify a biomarker panel consisting of a few genes whose expression patterns can indicate load stress states, a feature selection process was performed to select a reduced set of features capable of classifying load stress samples from the samples grown in other conditions. We applied four feature selection methods: RFE + RF, RFE + SVM, RGIFE + RF, RGIFE + SVM, which are two feature elimination strategies recursive feature elimination (RFE) (Guyon et al., 2002) and rank‐guided iterative feature elimination (RGIFE) (Lazzarini & Bacardit, 2017), in combination with a nonlinear classifier random forest (RF) or a linear classifier support vector machine (SVM).

RFE starts with an initial set of features and prunes a fixed number of least important features recursively until the desired number of features to select is eventually reached. The importance of each feature at each step is obtained based on the classifier model trained with the current set of features. Here the initial number of features is 4202. We set the number of features to eliminate at each step as two to make a trade‐off between computational costs and the ability to approach optimal solution.

RGIFE starts from a full feature set, and iteratively drops a dynamic block of features only if their removal has no negative effect on the predictive capacity of the current classifier model. The predictive performance of the model is estimated from stratified 10‐fold cross‐validation. The order in which features are checked for removal is determined by the ranking of feature importance produced by the classifier as part of its training process.

The feature elimination process here is adaptive. It removes a certain percentage of current features at each step, thus the number of features to be removed become increasingly smaller as the iteration progresses, resulting in a finer examination of the remaining features. The underlying rationale for this approach is that the features that are retained in the later steps are more likely to be relevant for the classification model. Here we set the percentage of features to be removed at each step as 25%. This means that when the current feature size reduces to six, which is the desired maximum feature size for this study, the model will evaluate the impact of each individual feature on the classification model.

If the trial to remove a block fails (because its removal led to a model of worse quality) the next block in order will be attempted. After five consecutive failed trials or all current features have been checked, the block size will be divided by 4 and the process will start again. Once the block size is reduced to 1, the algorithm will stop after five unsuccessful trials and will return the remaining features as the final panel, hence automatically deciding when to stop the iterative feature elimination process.

### 2.5 Determining the biomarker panel size and prioritizing the biomarker genes

To determine the optimal number of genes required in a biomarker panel, we repeatedly ran the aforementioned four feature selection models to obtain sufficient biomarker panels that vary in size for predicative capacity comparison. The desired number of features (biomarker genes) in RFE was set as 2–6 and each configuration was run for 100 times. For RGIFE, which automatically determines the final number of features to select, we first ran the model sufficiently to get various minimized feature sets that vary in size, and then retained those feature sets of size ranging from 2 to 6. Out of 1000 runs, only 22.8% of the biomarker panels identified by RGIFE consisted of more than six genes. Additionally, 7.2% of the panels consisted of less than 20 genes, while 10.3% consisted of more than 100 genes. We applied stratified 10‐folds cross‐validation to examine how the identified biomarker panels, as grouped by number of genes they consist of, would perform in terms of f1‐score.

To prioritize a few biomarker panels for further biological interpretation and evaluation, we assessed the identified biomarker panels using three criteria, that is, f1‐score, occurrence and margin. The occurrence was computed as the number of times a biomarker panel was selected by different models in multiple runs. The margin was computed as the minimal Euclidean distance between load stress samples and other samples in the hyperspace of biomarker gene expression. As shown in equation 3, **K** denotes a set of genes in a given biomarker panel, while $X_{I,K}$ and $X_{J,K}$ are the expression levels for biomarker genes (**K**) in load stress samples (**I**) and other samples (**J**) respectively. The margin can be negative when the expression changes of all biomarker genes between sample *i* and sample *j* showed the opposite trends than the mean expression changes between load stress samples and other samples.

(3) $$\begin{array}{ccl} {\mathrm{Margin}}_{K} & = & \min\left\{∥X_{i,K}-X_{j,K}∥\mathrm{sign}\left(i,j\right)|i\in I,j\in J\right\},\\ \mathrm{Sign}\left(i,j\right) & = & \left\{\begin{array}{ll} 1, & \;\text{if}\;\prod_{k}^{K}\;\text{sgn}\;\left(\bar{X_{I,k}}-\bar{X_{J,k}}\right)\;\text{sgn}\;\left(X_{i,k}-X_{j,k}\right)-1=0,\\ -1, & \;\text{otherwise}\;, \end{array}\right. \end{array}$$

## 3 RESULTS

### 3.1 Transcriptional response to load stress in *E. coli*

**Figure 1:**
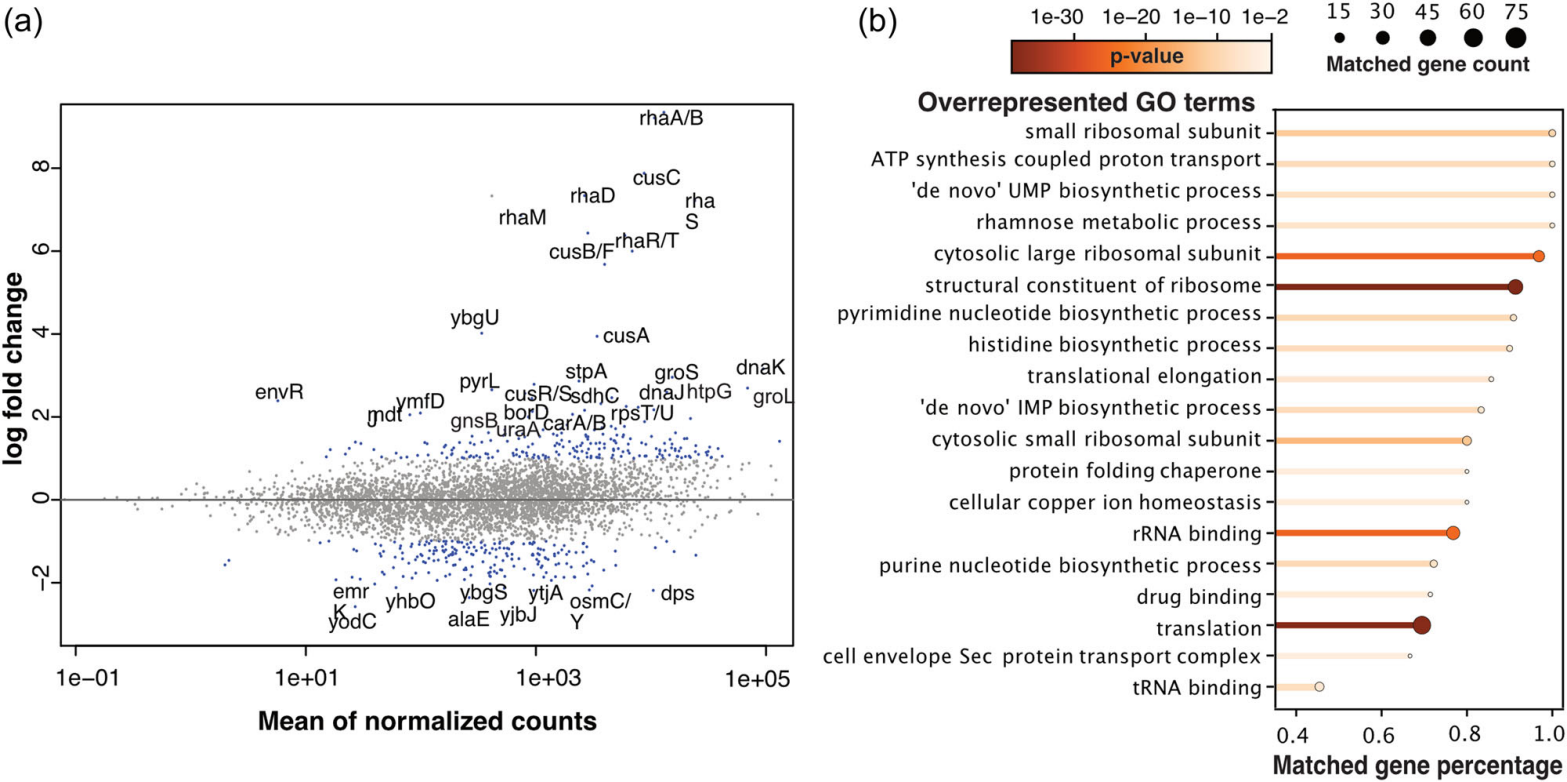
Transcriptional response to load stress in *Escherichia coli*. (a) The scatter plot shows the log fold change in read counts between wild‐type strain samples and engineered strains, as opposed to the mean read counts across all samples. The differentially expressed genes (DEGs) are highlighted in color blue (*p*‐value < 0.05, absolute log‐fold‐ change> (1) with top DEGs labeled (absolute log fold change> (2). (b) The overrepresented gene ontology (GO) terms are listed and ordered by “matched gene percentage,” that is, the percentage of genes in a given GO term that present in the list of DEGs. The color indicates the Fisher exact test *p*‐value, and the end dot size indicates the “matched gene count.”

The expression of heterologous genes in *E. coli* can place considerable burden on the host cell's growth and metabolic processes, initiating a complex set of stress responses termed load stress. To study the transcriptional response to load stress in *E. coli*, we compared the transcriptional profiles in heterologous gene expression samples with respect to wild‐type strain samples grown in control conditions (Figure 1). We detected 222 DEGs that were responsive to load stress and performed GO enrichment analysis to uncover common biological processes associated with these DEGs.

The top three overrepresented GO terms (as ranked by *p*‐values in Figure 1b) in the load stress state were “structural constituent of ribosomes,” “translation,” and “rRNA binding.” This explains part of the underlying mechanism of load stress, as overexpressing genes encoding recombinant proteins leads to a surge in mRNA production, causing a significant utilization of ribosomes for heterologous expression Glick (1995). We also found enrichment in pathways related to “ATP synthesis coupled proton transport” and “cell envelope Sec protein transport complex.” This is likely due to the excessive consumption of metabolic precursors such as amino acids, ATP, and NAD(P)H (Ramchuran et al., 2005) as well as the toxic effects of inducers like isopropyl β‐D‐1‐thiogalactopyranoside (IPTG) (Dvorak et al., 2015) and the replication of plasmids or the expression of resistance genes (Mairhofer et al., 2013; Silva et al., 2012).

**Figure 2:**
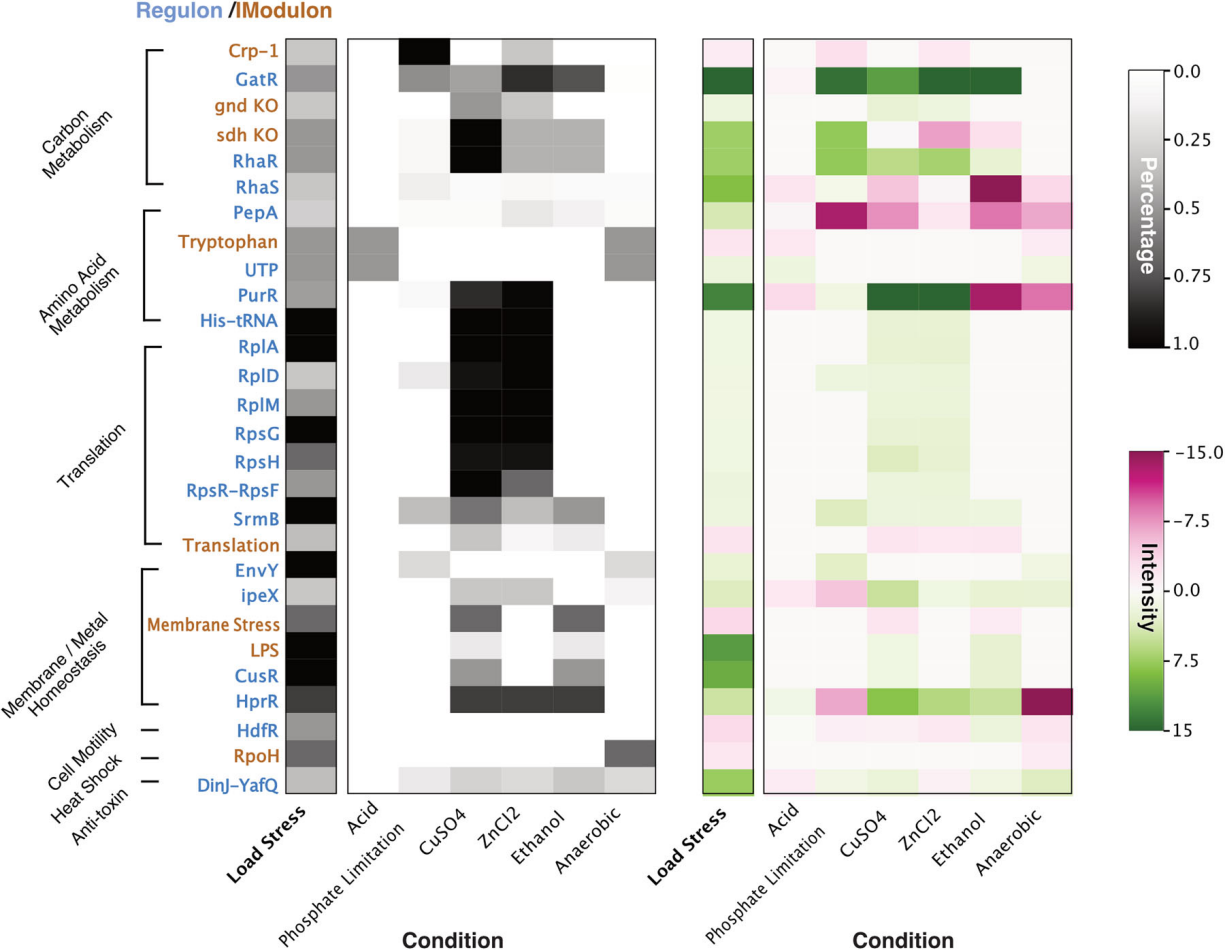
The regulons and iModulons activities in response to load stress as compared to other conditions. The heatmap displays the activity levels of each regulon or iModulon in terms of percentage index and intensity index respectively. Here we present only the regulators and iModulons that exhibited varied activity levels (percentage index > 0.3) in response to load stress. The regulon names are displayed in blue, while the iModulon names are displayed in brown.

We further analyzed the activities of transcriptional regulators and iModulons in the load stress response as compared to 52 different environmental changes (Supporting Information: Table S2b,c). A subset of regulons or iModulons showed varying activity levels in heterologous expression samples (Figure 2), among which some were known to be involved in the stress responses of *E. coli*. For instance, we found that the expression of ribosomal components *rpsGHRF* and *rplADM* was modulated, which helped to meet the elevated requirement for ribosomes during heterologous expression (Haddadin & Harcum, 2005). The upregulation of the UTP regulon (consisting of genes *pyrE*, *pyrL*, *pyrI*, and *pyrB*) played a vital role in pyrimidine biosynthesis, which is essential for the synthesis of DNA and RNA (Bonekamp et al., 1984). By upregulating these genes, the cells were able to produce more mRNA and DNA precursors for expressing the recombinant proteins. These findings are consistent with the above GO enrichment analysis results.

Additionally, we observed the modulation of DinJ‐YafQ regulon, a well‐studied toxin–antitoxin (TA) system in *E. coli*. TA systems, consisting of a pair of closely linked toxin gene and antitoxin gene, are known to be associated with bacterial stress response, programmed cell death, and maintenance of plasmids. Here, the *dinJ* gene encodes for a toxin and its adjacent gene, *yafQ*, encodes for the corresponding antitoxin. The toxin DinJ functions by inhibiting translation or mRNA synthesis, leading to a slowdown in cellular growth and potentially triggering a dormant state (Motiejūnaitė et al., 2007). As an evolutionary survival strategy that allows bacteria to persist in adverse conditions, the DinJ‐YafQ system was thus induced under various stress conditions (Figure 2), including load stress, anaerobic growth, and nutrient limitation.

Also of note was the upregulation of RpoH sigma factor in load stress and anaerobic condition (Figure 2). It is known that the activation of the rpoH gene in *E. coli* leads to increased production of σ32, which triggers the expression of heat shock proteins for recovery from heat‐induced damage. A prior study also demonstrated the activation of rpoH during load stress and adopted heat shock genes as load stress reporters (Ceroni et al., 2018). Meanwhile, we observed the modulation of EnvY and IpeX regulons, which are associated with membrane homeostasis, in load stress as well as in exposure to CuSO_4_, ZnCl_2_, Ethanol. This suggests a more complex transcriptional changes in load stress response that are overlapped with other stress response processes.

Last, the load stress samples exhibited an overrepresented GO term “rhamnose metabolic process” and increased transcriptional activity in regulators RhaR and RhaS. This effect can be attributed to the use of rhamnose as an inducer in all heterologous gene expression samples. As a result, the relevant genes may not be suitable candidate biomarkers for specifically sensing the transcriptional changes induced by load stress.

### 3.2 Pairs of genes can serve as transcriptional biomarkers to sense load stress

**Figure 3:**
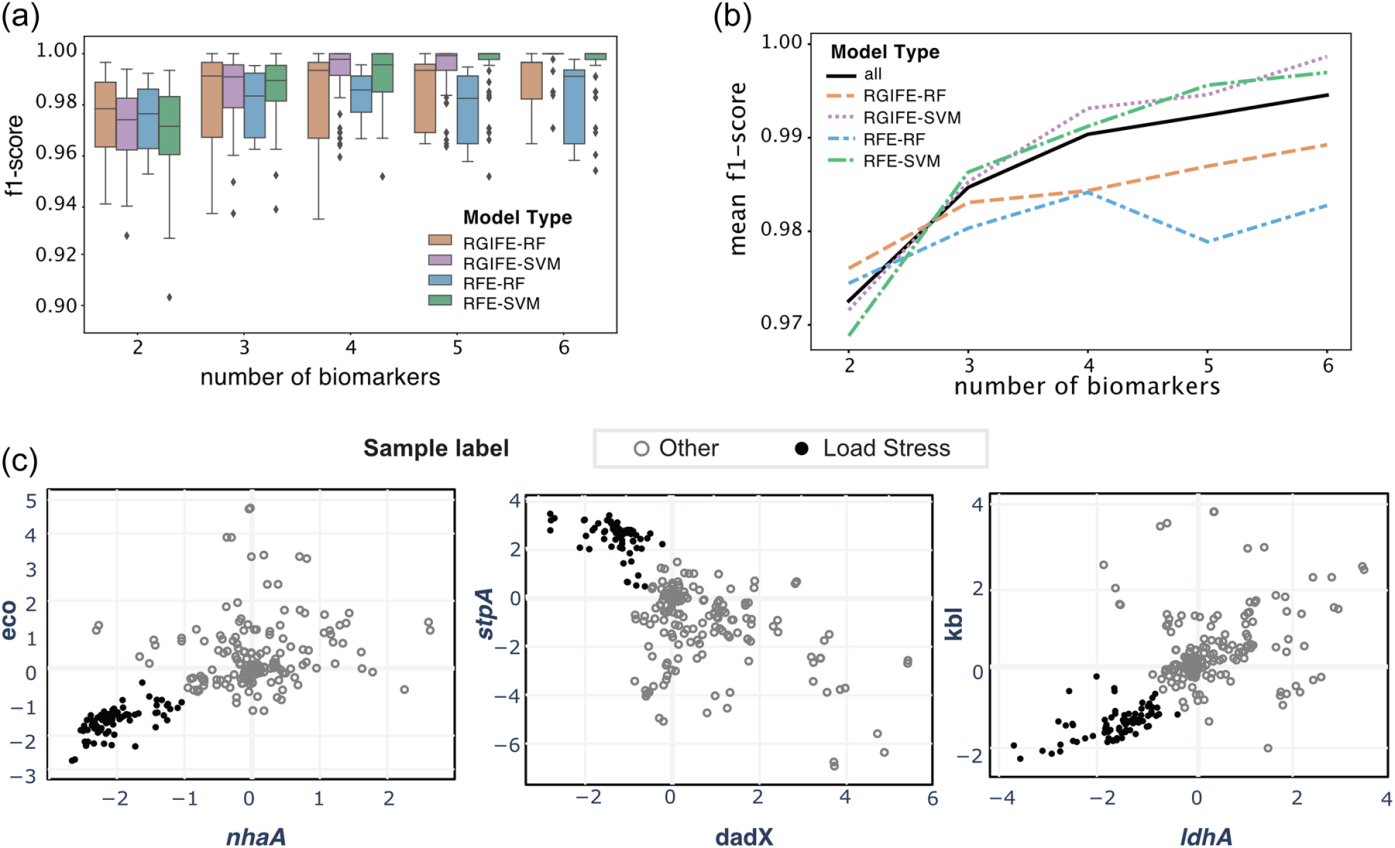
The performance of various biomarker panels to predict load stress in *Escherichia coli*. (a) The performance distribution across 100 repetitions of cross‐validation tests for biomarker panels selected by four different models and of varying sizes (from 2 to 6). (b) The mean performance for each of four models and all models combined. (c) The gene expression levels of three pairs of identified biomarker genes are shown in the scatterplots. The black circle markers represent the samples from the load stress group while the gray open circle markers represent the other samples grown in various conditions.

We performed the feature selection process repeatedly on training folds of data with four different models and estimated the overall performance by using the selected biomarker genes to predict the test folds of data. We obtained the cross‐validation f1‐scores for 2291 biomarker panels of size varying from 2 to 6 and by four models (Figure 3a). RFE‐SVM and RGIFE‐SVM embedded with SVM classifier performed better for selecting panel size larger than 2. RIFESS‐RF and RGIFESS‐SVM adopting dynamic feature elimination strategy performed better than RFE‐RF and RFE‐SVM in general.

The overall performance for all models except RFE‐RF improved as the biomarker panel size increased (Figure 3b). However, building more biomarker genes in the load stress reporter system can add challenges and costs in the design of the system. We found that the biomarker panels consisting of two or three genes are able to discriminate load stress samples with median f1‐score at 0.972 and 0.984, respectively.

We reported 13 biomarker panels consisting of two genes that achieved great predictive power (f1‐score >0.98, margin >1) in Table 1, from which we highlighted in bold font (a) a biomarker pair with the highest occurrence which was most stable across multiple runs of different biomarker identification methods, (b) a biomarker pair with the highest f1‐score which most accurately predicated load stress states, and (c) a biomarker pair with the highest margin which showed the largest effect size to discriminate the load stress samples from other samples. The gene expression patterns of these biomarker pairs in load stress samples were distinct from the other samples (Figure 3c), and thus can be coupled to build synthetic systems capable of predicting load stress.

**Table 1:** The shortlist of biomarker pairs.

Biomarker genes	Model names	Occurrence	f1‐score	Margin
***nhaA;eco***	RGIFE‐RF;RFE‐RF	32	0.993	**0.397**
*eco;queE*	RGIFE‐RF;RFE‐RF	13	0.987	0.293
***nhaA;yhcN***	RGIFE‐SVM;RFE‐SVM	4	0.989	0.276
*yodC;ygbA*	RGIFE‐SVM;RFE‐SVM	9	0.984	0.216
*gnsB;pabA*	RFE‐SVM	1	0.993	0.200
***ldhA;kbl***	RGIFE‐SVM	10	0.997	0.195
*cusC;stpA*	RGIFE‐SVM	6	0.994	0.194
*nhaA;mdlA*	RGIFE‐RF	1	0.996	0.192
***dadX;stpA***	RGIFE‐RF;RFE‐RF	**40**	0.992	0.184
*pqiA;eco*	RGIFE‐RF	12	0.991	0.165
*nhaA;aaeA*	RGIFE‐RF	1	0.991	0.140
*aaaD;ygbA*	RFE‐SVM	1	0.987	0.124
*dadX;ldhA*	RGIFE‐RF	1	0.987	0.110

*Note*: These bold values (Occurrence: 40; f1‐score: 0.997; Margin: 0.397) are to highlight the correpsonding biomarker genes that excel by three different ways of assessments.

### 3.3 Biological characterization of the biomarker genes

**Figure 4:**
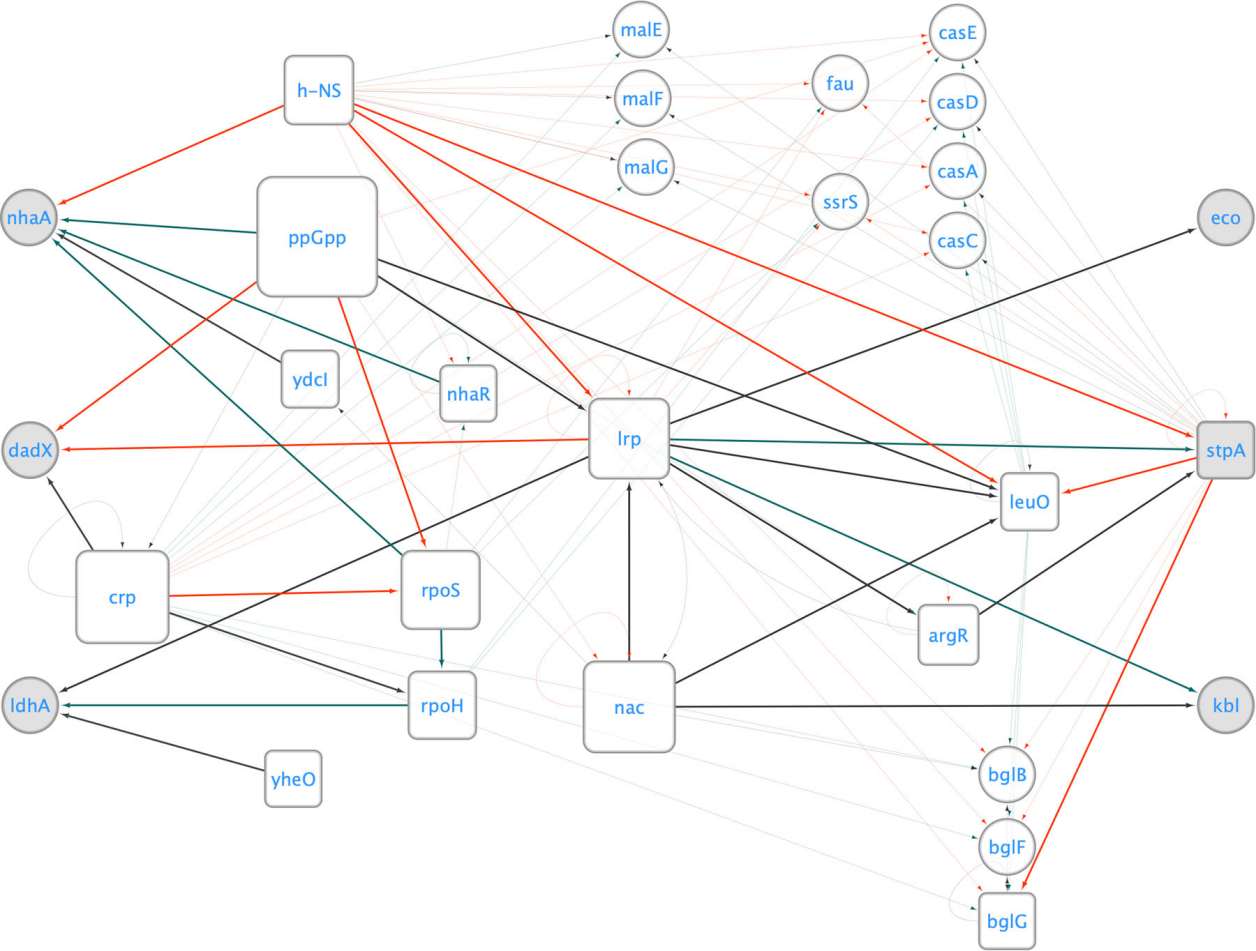
A subnet of *Escherichia coli* transcriptional regulatory network pertained to three load stress biomarker pairs. All the shortest paths connecting any two biomarker genes as well as the regulatory paths directly related to biomarkers are highlighted in bold stroke. The node rpoD, which regulates more than 1500 genes, is removed from this network, despite its association with the biomarker genes. The genes encoding regulators or regulon names (h‐NS) are plotted as squares, while the regulated genes are plotted as circles. The biomarker genes are highlighted in color gray. The red edge indicates activation, the green edge indicates repression, and the dark gray edge indicates that the interactions.

To explore the biological relevance of the three highlighted biomarker pairs, we first investigated their individual products and functions. As listed in Table 2, the biomarker genes showed downregulation under load stress as compared to other conditions are indicated in color red, while the upregulated biomarker genes are displayed in color green. We then studied the transcriptional factors that are directly or indirectly associated with the biomarkers and the chains of transcriptional activities between them. This is presented by extracting a smallest component from *E. coli* Gene Regulatory Network that connects these six biomarker genes (Figure 4).

**Table 2:** Gene annotations for three highlighted *E. coli* load stress biomarker pairs

	Gene	Product	Clusters of orthologous genes
Pair 1	*nhaA*	Na(+):H(+) antiporter NhaA	Inorganic ion transport and metabolism
	*eco*	Serine protease inhibitor ecotin	Cell wall/membrane/envelope biogenesis
Pair 2	*dadX*	Alanine racemase 2	Amino acid transport and metabolism
	*stpA*	DNA‐binding transcriptional repressor	Transcription
Pair 3	*kbl*	2‐amino‐3‐ketobutyrate CoA ligase	Coenzyme transport and metabolism
	*ldhA*	d‐lactate dehydrogenase	Energy production and conversion

In the first biomarker pair, the gene *nhaA* is responsible for encoding an Na^+^:H^+^ antiporter, playing an essential role in regulating sodium ion and alkaline pH levels within cells. Previous study has showed that NhaA also has a significant role in sustaining antibiotic tolerance during periods of starvation (Wan et al., 2021). The gene *eco* encodes ecotin working as an inhibitor for trypsin and a number of additional heterologous proteases. As revealed by a study of ecotin's interaction with mannan‐binding lectin‐associated serine proteases (MASPs), ecotin can act as a defense factor against microbes, and thus could be a promising target for antibiotic development (Nagy et al., 2019).

In the second biomarker pair, the gene *dadX* encodes a type II alanine racemase that catalyzes the interconversion of D‐ and L‐alanine. Overexpression of a DadX K35A Y253A double mutant have shown resulting in growth inhibition (Strych & Benedik, 2002). The gene *stpA* encodes a DNA‐binding transcriptional repressor that has similarity functions with H‐NS (Shi & Bennett, 1994). It can block the access to DNA by forming a rigid filament and has a preference for curved DNA (Sonnenfield et al., 2001).

In the third biomarker pair, the gene *kbl* encodes 2‐Amino‐3‐ketobutyrate CoA ligase which involved in the second reaction in the threonine dehydrogenase‐initiated pathway, where threonine is converted to glycine and consequently serine. This pathway is the primary route for threonine utilization and is an alternate pathway for serine biosynthesis in *E. coli* Sharma et al. (2011). The gene *ldhA* encodes a soluble NAD‐linked lactate dehydrogenase (LDH) for producing D‐lactate specifically. The expression of *ldjA* is repressed by regulator ArcA (Shalel‐Levanon et al., 2005), which controls the carbon oxidation. Although *ldhA* is regulated by RpoH and belongs to the 32 regulon, there have been arguments on whether it is induced by heat shock (Hasan & Shimizu, 2008).

While more than two biomarker genes are commonly regulated by the global transcriptional factors Lrp, RpoD, ppGpp, or h‐NS, the biological enrichment analysis in this biomarker‐induced gene regulatory network did not reveal any underlying mechanisms shared by these biomarker genes. It is important to note that each pair of genes was selected as a biomarker set based on the combination of their gene expression patterns. That is, these pairs of genes provide complementary information that together have good discriminative power. Hence, these genes are most likely to be functionally orthogonal and not necessarily related, presumably to be involved in different pathways. While the expression pattern of a single biomarker gene may not be unique to load stress, along with another biomarker expression characteristic a unique expression fingerprint is provided.

**Figure 5:**
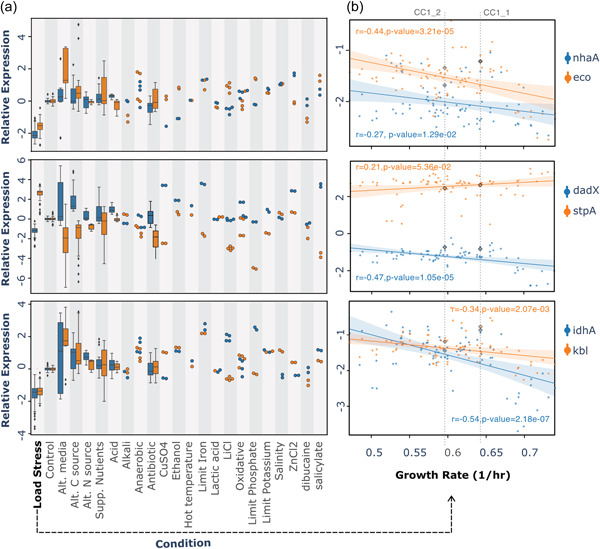
The expression levels of load stress biomarkers and the growth rates of the engineered *Escherichia coli* strains. (a) The expression levels of biomarker genes are represented as jittered marks or bar plots (if there are more than eight data points) in strip plots. Each column shows the gene expression in a different condition, with the load stress in the first column as compared to various other conditions in the rest 22 columns. Two genes in a biomarker pair are discriminated in color blue and orange. (b) The scatter plots with the regression lines show the gene expression levels of three biomarker pairs against the cellular growth rates in load stress samples. The Pearson correlation coefficients and corresponding p‐values are provided to quantify the strength of the linear relationship. Two genes in a biomarker pair are discriminated in color blue and orange. Samples inserted with empty plasmid (CC1_1, CC1_2) are marked with black diamond symbols.

To further characterize the transcriptional behaviors of the identified biomarker genes, we compared the expression levels of the biomarker genes in load stress samples with their expression in other samples grown in various conditions. As shown in Figure 5a, the relative expression levels reflect the transcriptional changes incurred by moving the samples to 23 treatment condition from the corresponding control conditions. We found that at least one gene in a biomarker pair presented the opposite trend or much larger scale of the same trend of transcriptional changes in response to load stress as against most of the other conditions. However, multiple individual biomarker genes saw similar transcriptional changes in some stimuli (such as Dibucaine, CuSO_4_, and Alkali) as in load stress, suggesting that common stress response mechanism may be shared.

As the load stress can be induced by expressing different synthetic constructs in *E. coli* samples and affecting the cellular growth rate, these samples may suffer from different levels of load stress and reduced growth. We explored the relationships between biomarker gene expression and cellular growth rate in 82 load stress samples (Figure 5b). The pair of samples with empty plasmid inserted (marked by diamond symbols in Figure 5b) showed moderate degradation in growth rate and were considered under load stress as well. We noticed a reverse correlation between the expression of all the downregulated biomarkers (i.e., *nhaA*, *eco*, *dadX*, *idhA*, *kbl*) and the growth rate in load stress samples (with *p* < 0.05). The expression of the sole upregulated biomarker, the gene *stpA*, exhibited a general upward trend as the growth rate increased, although it is important to note that this correlation did not reach statistical significance. These observations imply that there may be a relationship between the extent of modulation in biomarker expression in response to load stress and the resulting growth rate. In other words, the growth rate in load stress samples may be more significantly impacted when there is minimal modulation of these biomarker genes.

## 4 DISCUSSION

The expression of synthetic constructs for heterologous protein production in host bacteria can lead to resource and energy competition (Gyorgy & Del Vecchio, 2014; Kastberg et al., 2022), triggering a load stress response and compromising the performance of the constructs. The goal of this study was to identify the genes that were uniquely responsive to load stress in *E. coli*, so as to enable effective engineering strategies for relieving the cellular load stress and stabilizing the heterologous protein production. This was achieved by designing computational methods to exploit a RNA‐seq data set that measured the gene expression profiles of noninduced *E. coli* cells with diverse environmental perturbations and induced *E. coli* cells with a large set of heterologous genes inserted.

The differential expression analysis showed that complex transcriptional adaptions occurred in the context of load stress, which were related to various biological processes such as carbon and Amino Acid Metabolism, Translation, Membrane Homeostasis, Heat Stress, and Anti Toxin. We performed an ensemble of feature selection methods to minimize the number of genes required to discriminate load stress state from normal and other stress states, avoiding the selection of some genes over others as biased by certain classifier or feature elimination manner. We found that pairs of genes can already predict load stress samples with accuracy higher than 0.97.

This study is subject to several limitations. First, our identification of load stress biomarkers relies on a single RNA‐seq data set, wherein the engineered *E. coli* strains shared the same plasmid design. Future work should extend our methods to encompass a broader spectrum of synthetic constructs from additional datasets. This expansion would enable the selection of biomarkers capable of representing a more diverse range of load stress conditions. Second, we must acknowledge that we could not conduct additional experimental verification of the reported biomarker genes. The validation of these biomarker genes' effectiveness in sensing well‐defined load stress states is a crucial area of future research. Moreover, there remains substantial scope for further investigation into the relationships between biomarker expression levels, host cell growth rates, and the expression levels of synthetic constructs.

Our biomarker gene pairs can be readily incorporated in the most recent burden feedback system from Ceroni et al. (2018) to make load stress reporting system presumably with improved specificity. This is because the biomarker genes identified in this work were unique to load stress—they can discriminate the load stress from diverse cellular states that might be triggered in other stress conditions. Moreover, “live cell” biosensors that adopt single‐cell technologies such as flow cytometry (Heins et al., 2021) or microfluidics (Dusny & Grünberger, 2020; Sampaio et al., 2022) can also be potentially adapted for sensing load stress using our biomarker genes.

As this work only studied a single RNA‐seq data set where the engineered *E. coli* strains used same plasmid design, it is preferred to perform validation of our methods on additional synthetic constructs expression from independent samples. Future work also includes the study of the correlation between load sensing gene expression levels and host cell growth rates as well as synthetic construct expression levels.

In this work, we provided a quantitative approach to identify pairs of gene signatures that can accurately predict load stress state induced by synthetic construct expression with respect to other cellular states. The biomarker genes identified in this work can be further applied to build a load stress reporting system.

## AUTHOR CONTRIBUTIONS

*Conceptualization*: Yiming Huang, Jaume Bacardit, and Anil Wipat. *Data curating and methodology*: Yiming Huang. *Formal analysis, software, and visualization*: Yiming Huang. *Validation*: Yiming Huang, Jaume Bacardit, and Anil Wipat. *Investigation*: Yiming Huang and Jaume Bacardit. *Resources*: Yiming Huang, Jaume Bacardit, and Anil Wipat. *Writing—original draft preparation*: Yiming Huang. *Writing—review and editing*: Yiming Huang, Jaume Bacardit, and Anil Wipat. *Supervision*: Jaume Bacardit and Anil Wipat. *Funding acquisition*: Jaume Bacardit and Anil Wipat. All authors have read and agreed to the published version of the manuscript.

## CONFLICT OF INTERESTS STATEMENT

The authors declare no conflict of interest.

## Supporting information

Supplementary Information

Supplementary Information

## Data Availability

Data used in this work can be downloaded at https://github.com/SBRG/precise2.
